# Secukinumab on Refractory Lupus Nephritis

**DOI:** 10.7759/cureus.17198

**Published:** 2021-08-15

**Authors:** Rita Costa, Paula Antunes, Pedro Salvador, Pedro Oliveira, António Marinho

**Affiliations:** 1 Internal Medicine Department, Centro Hospitalar Vila Nova De Gaia, Vila Nova de Gaia, PRT; 2 Internal Medicine, Hospital de Cascais Dr José de Almeida, Lisboa, PRT; 3 Internal Medicine, Centro Hospitalar do Porto, Porto, PRT

**Keywords:** systemic lupus erythematosus, lupus nephritis, il-17 inhibitor therapy, secukinumab, proteinuria, th17 cells, cytokines, immunosuppressive therapy

## Abstract

Lupus nephritis (LN) is the most frequent severe organ manifestation of systemic lupus erythematosus (SLE). About 30% of patients are refractory to treatment. The authors report a case of treatment of LN with interleukin-17-targeted therapy, demonstrating its possible benefit, after reports of T helper 17 cell involvement in SLE pathogenesis.

We present the case of a childbearing age woman with SLE, who developed refractory LN despite all the indicated therapeutic options. During follow up, infection with human papillomavirus was detected, a possible trigger, and the following management was based on this discovery. We currently know that cytokines play a major role in tissue damage and interleukin-17 (IL-17) seems to be a fundamental key in SLE and LN, having shown its expression in renal glomeruli and urinary sediment. Thus, it was decided to start treatment with an anti-IL-17A antibody, secukinumab. After starting secukinumab, clinical and biological features improved and complete renal response was achieved.

## Introduction

Systemic lupus erythematosus (SLE) is an autoimmune disorder characterized by chronic inflammation, which can result in a multitude of systemic or organ-limited involvement, including the skin, lungs, heart, and kidney. The kidney is the most frequent organ affected in SLE, with various grades of severity [1,2]. Nephritis affects 30-60% of adults and up to 70% of children with SLE. Immune complex deposition in various parts of the glomerulus defines the several classes of lupus nephritis (LN), based on the 2003 International Society of Nephrology and the Renal Pathology Society classification scheme. LN should be immediately thought of in the presence of proteinuria.

Accordingly to the European Alliance of Associations for Rheumatology (EULAR) recommendations, initial (induction) therapy should consist of glucocorticoids combined with either cyclophosphamide (CY) or mycophenolate mofetil (MMF) in an attempt to induce partial or complete renal remission.

Once a patient has attained a complete or partial response, immunosuppression is continued to help maintain the response and decrease the risk of developing end-stage renal disease. The two most commonly used drugs for maintenance therapy are MMF and azathioprine. About 30% of patients present with refractory disease/non-responding [3]; in this group treatment options include switching between CY and MMF, or addition of rituximab, the latter potentially in combination with belimumab [1]. Less evidence supports extracorporeal treatment (plasma exchange or immunoadsorption), calcineurin inhibitors (cyclosporine A or tacrolimus), intravenous immunoglobulin and stem cell transplantation [1].

We present the case of a childbearing age woman with SLE who developed refractory LN. During follow up, infection with human papillomavirus was detected. It was decided to start treatment with an anti-interleukin-17A antibody, secukinumab. After starting secukinumab, clinical and biological features improved and complete renal response was achieved. The authors report a case suggesting the efficacy of IL-17A inhibitor therapy in LN.

## Case presentation

A 29-year-old woman was diagnosed with SLE in 2011, presenting with severe arthritis (polyarthritis of large and small joints), Raynaud's phenomenon, photosensitivity, malar rash and severe scarring alopecia. The initial blood tests showed high inflammatory parameters (C-reactive protein and erythrocyte sedimentation rate), complement consumption (C3 and C4), as well as anti-double stranded DNA (anti-dsDNA) > to 400 IU/ml (ELISA immunoassay). She started initial pulse intravenous methylprednisolone, followed by 10mg of oral prednisolone and hydroxychloroquine 400mg per day, with clinical and analytical improvement for a short period.

At the beginning of the disease, due to severe arthritis flares, she was on azathioprine and finally methotrexate was added with complete response. In 2014, due to cutaneous flare with severe discoid rash, she was started on thalidomide with complete response that lasted for three years, when she had a new articular flare. Relapsing-remitting disease without permanent low disease activity was assumed and off-label rituximab was prescribed (1g x 2 in July 2017). There was a global improvement in lupus activity.

In September 2017, after rituximab, she developed a nephritic syndrome requiring hospitalization. Class IV lupus nephritis was confirmed by biopsy (interstitium with inflammatory infiltrate, predominantly lymphomononuclear and minimal fibrosis, with additional tubular atrophy - less than 5% of cortical extension; direct immunofluorescence study revealed glomerular deposits in the basement membrane for C3, C4, C1q, IgA, IgG and IgM). Hospitalization was complicated by seizures due to malignant hypertension. In this phase (September 2017), she started mycophenolate mofetil in an induction scheme reaching 3g per day.

In April 2018, partial renal remission was assumed despite maintaining proteinuria in a range close to nephrotic but presenting normal glomerular filtration rate (estimated with CKD-EPI about 115mL/min/1,73m2). However, she persisted with severe general symptoms and severe diffuse discoid rash. Thus, it was decided to start a new cycle of rituximab (1g x 2) and reduction of mycophenolate mofetil as a second induction scheme for lupus nephritis. Yet she stayed unresponsive and we added on immunoglobulin cycles and finally intravenous belimumab.

In February 2019 the patient kept the severe mucocutaneous component and relapsed nephritis, clearly showing unresponsiveness to belimumab. Analytical data evolution can be consulted in Table 1.

**Table 1:** Data of the analytical parameters RV- reference value

Parameter (RV and units)	Dx 2011	05/2014	07/2017	09/2017	03/2018	04/2018	02/2019	04/2019	10/2019	03/2020	06/2020
Erythrocyte sedimentation rate (0-19 mm)	54	33	51	52	35	15	28	25	14	18	17
Reactive C Protein (0-5, mg/L)	0,61	4,12	10,68	5	0,72	---	1,08	0,49	0,95	0,82	0,8
C3 (81-167 mg/dL)	31	103	53,2	50,8	96,3	118,8	114,6	121,5	100,4	112,4	95,8
C4 (12-42 mg/dL)	<2	15	4,4	7,7	22,7	23,7	24,3	24	23,2	30,6	32,8
Anti-double stranded DNA (< 15 UI/mL)	> 400	58	> 400	153	85	87	55	48	33	30	32
Creatinine (0,5-0,9 mg/dL)	---	0,85	0,75	1,19	0,74	0,72	0,67	0,62	0,66	0,64	0,67
Urea (70-105 mg/dL)	---	47	28	54	46	48	30	49	75	70	30
Seric albumin (3,5-6 g/dL)	3,73	4,77	3,95	2,65	4,02	4,34	4,12	---	3,61	4,32	---
Proteinuria in 24 hours (0,03- 0,15 g/dia)	---	---	---	---	2,35	2,50	1,86	---	2,16	0,79	0,34
Urinary sediment	Innocent	Innocent	Erythrocyturia	Erythrocyte dysmorphia	Erythrocyturia	Erythrocyturia	Erythrocyturia	Erythrocyturia	Inadequate sample	Innocent	Innocent

During the course of the disease, a possible viral agent was identified as a trigger, the human papillomavirus (HPV). At this time it was decided to suspend mycophenolate mofetil and start an agent with possible anti-viral effects and possible benefit in lupus nephritis. Thus, she was started on leflunomide (initial loading dose of 100mg daily for three days, followed by 20mg daily). It was decided to suspend belimumab in April 2019.

Despite all the choices of treatment, the patient remained refractory to treatment. Given the association with HPV infection, it was decided to start secukinumab (300mg subcutaneously), an anti-interleukin 17 antibody, in October 2019.

**Figure 1:**
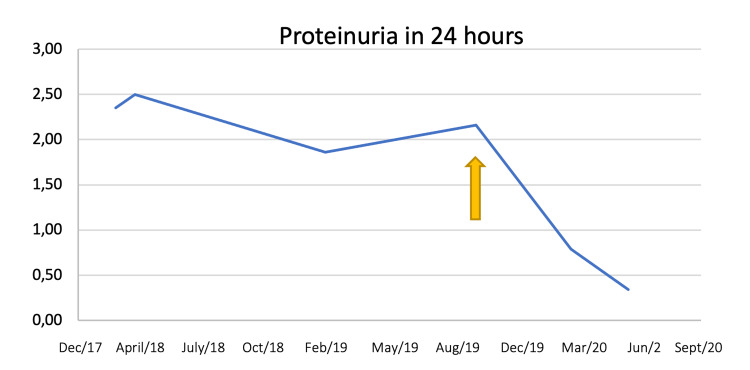
Proteinuria evolution over time. The yellow arrow indicates the timing of secukinumab introduction.

In five months of treatment almost partial renal response was achieved, with proteinuria 760mg per 24 hours. Complete renal response criteria was obtained in June 2020, eight months after starting treatment (Figure 1).

## Discussion

Some patients with LN do not respond to conventional treatment with immunosuppressive drugs or experience recurrence. Understanding the etiopathogenesis of LN could help to direct treatment and prevent progression to end-stage renal failure. Multiple immunological pathways are involved in inducing tissue damage in SLE. Cytokines are key mediators in this process and among them the role of interleukin (IL)-23/Th17 axis has recently emerged, as IL-17 and Th17 cells seem to play a major role in the pathogenesis of both SLE and LN [2,4,5]. The involvement of these cytokines was described by various authors in mouse and also in human lupus models [2,6-11].

Th17 cells can contribute to several pathological pathways of SLE, such as the induction of vascular inflammation, recruitment of leukocytes, activation of B cells and autoantibody production [12], contributing to glomerular injury and to the persistence of inflammation and renal damage [9,10].

Several studies showed that IL-17 production and IL-17-producing CD4+ and CD3+ CD4-CD8- cells are increased in patients with SLE [9,11,13]. Also, a strong correlation between the frequency of CD4+ IL-17+ T cells and disease activity in SLE patients has been observed [14-16].

Regarding LN, higher levels of glomerular IL-17 and IL-23 expression are observed in renal biopsies [9] from class IV LN patients as compared with those from minimal change nephropathy patients and normal controls. Both glomerular IL-17 and IL-23 expression levels positively correlate with renal histological activity index scores for LN patients [14].

High IL-17 serum levels were shown to be a marker of adverse histopathological outcome after immunosuppressive therapy in LN, indicating that IL-17 production could be associated with a severe or therapy-resistant disease phenotype [13].

The regulatory role of TH17-related cytokines in pathogenesis of LN has also been demonstrated by examining the gene expression of TH17-related cytokines in the urinary sediment of SLE patients [17]. It has been described, a higher urinary expression of TH17-related cytokines in SLE patients as compared with healthy controls. The degree of up-regulation of TH17 related genes is inversely related to both systemic and renal lupus activity, as well as urinary expression of TH1-related genes. Urinary expression TH17-related genes increase again following successful immunosuppressive treatment of active disease [17].

Th17 cells exhibit a high degree of plasticity, which means CD4+ IL-17 producing T cells can potentially acquire features of Th1 or, alternatively, T regulatory (reg) cells. Due to their opposite effects on the immune response, Th17/Treg balance is critical in maintaining immune homeostasis, and if Treg cells are defective, they can be converted into Th17 cells leading to inflammatory diseases [12].

Zickert et al. analyzed 52 patients with LN and acknowledged that high baseline levels of IL-17 acted as a marker of poor histopathological and clinical outcome after immunosuppressive therapy, suggesting that high IL-17 production could be associated with a severe, or therapy-resistant, disease phenotype. In this same study, immunostaining revealed IL-17 expression in renal tissue from LN patients, most pronounced in areas with infiltrating T-cells, this confirms the role of IL-17 in the inflammatory process in the renal tissue [18].

The concept of inhibition of the IL-17 pathway as a therapeutic option has advanced into clinical trials in SLE, with the IL-12/IL-23 inhibitor ustekinumab (Stelara) targeting the IL-17 pathway currently being evaluated in a Phase III clinical trial in controlling disease activity of moderate-to-severe SLE [19].

Regardless of all the knowledge data suggesting the potential benefits of this drug in LN, there is another feature that led to the choice of treatment in the patient mentioned: its action on HPV. It has been proved that anti-IL-17 therapy decreases HPV detection [20], although the mechanism had not been fully understood, research found that elevated IL-17 was associated with inhibited effective host immune responses against HPV [21].

## Conclusions

Some patients with severe LN, despite immunosuppressive treatment, will deteriorate leading to end-stage renal disease. Thus, it is emergent to develop a treatment modality more effective for LN patients. Our experience with this case suggested that there is a group of patients with SLE who are highly resistant to conventional treatment in which pathogenic Th17 cells play a major role in pathogenesis, and that IL-17 inhibitor therapy may be a new effective treatment option for such patients.

Given the important heterogeneity in terms of clinical manifestations and SLE severity, the potential beneficial effect of IL-17 blockade may be limited to a subpopulation of patients whose disease is predominantly promoted by the IL-17 pathway. Therefore, it is crucial to identify biomarkers that can be used for patient screening to identify responders to IL-17 therapy.

## References

[1] Kronbichler A, Brezina B, Gauckler P, Quintana LF, Jayne DR (2019). Refractory lupus nephritis: when, why and how to treat. Autoimmun Rev.

[2] Koga T, Ichinose K, Kawakami A, Tsokos GC (2019). The role of IL-17 in systemic lupus erythematosus and its potential as a therapeutic target. Expert Rev Clin Immunol.

[3] Atisha-Fregoso Y, Malkiel S, Harris KM (2021). Phase II randomized trial of rituximab plus cyclophosphamide followed by belimumab for the treatment of lupus nephritis. Arthritis Rheumatol.

[4] Farah Izati A, Wong KK, Che Maraina CH (2020). IL-23/IL-17 axis in the pathogenesis and treatment of systemic lupus erythematosus and rheumatoid arthritis. Malays J Pathol.

[5] Wong CK, Lit LC, Tam LS, Li EK, Wong PT, Lam CW (2008). Hyperproduction of IL-23 and IL-17 in patients with systemic lupus erythematosus: implications for Th17-mediated inflammation in auto-immunity. Clin Immunol.

[6] Amarilyo G, Lourenço EV, Shi FD, La Cava A (2014). IL-17 promotes murine lupus. J Immunol.

[7] Martin JC, Baeten DL, Josien R (2014). Emerging role of IL-17 and Th17 cells in systemic lupus erythematosus. Clin Immunol.

[8] Apostolidis SA, Crispín JC, Tsokos GC (2011). IL-17-producing T cells in lupus nephritis. Lupus.

[9] Crispín JC, Oukka M, Bayliss G (2008). Expanded double negative T cells in patients with systemic lupus erythematosus produce IL-17 and infiltrate the kidneys. J Immunol.

[10] Zhang Z, Kyttaris VC, Tsokos GC (2009). The role of IL-23/IL-17 axis in lupus nephritis. J Immunol.

[11] Pisitkun P, Ha HL, Wang H (2012). Interleukin-17 cytokines are critical in development of fatal lupus glomerulonephritis. Immunity.

[12] Larosa M, Zen M, Gatto M (2019). IL-12 and IL-23/Th17 axis in systemic lupus erythematosus. Exp Biol Med (Maywood).

[13] Yanti T, Yuliasih Yuliasih, Rahmawati LD (2020). IL-23/IL-17 axis and disease activity in systemic lupus erythematosus patients. Eurasia J Biosci.

[14] Chen DY, Chen YM, Wen MC, Hsieh TY, Hung WT, Lan JL (2012). The potential role of Th17 cells and Th17-related cytokines in the pathogenesis of lupus nephritis. Lupus.

[15] Talaat RM, Mohamed SF, Bassyouni IH, Raouf AA (2015). Th1/Th2/Th17/Treg cytokine imbalance in systemic lupus erythematosus (SLE) patients: correlation with disease activity. Cytokine.

[16] Shah K, Lee WW, Lee SH, Kim SH, Kang SW, Craft J, Kang I (2010). Dysregulated balance of Th17 and Th1 cells in systemic lupus erythematosus. Arthritis Res Ther.

[17] Kwan BC, Tam LS, Lai KB (2009). The gene expression of type 17 T-helper cell-related cytokines in the urinary sediment of patients with systemic lupus erythematosus. Rheumatology (Oxford).

[18] Zickert A, Amoudruz P, Sundström Y, Rönnelid J, Malmström V, Gunnarsson I (2015). IL-17 and IL-23 in lupus nephritis - association to histopathology and response to treatment. BMC Immunol.

[19] (2021). A Study of Ustekinumab in Participants With Active Systemic Lupus Erythematosus. https://clinicaltrials.gov/ct2/show/study/NCT03517722.

[20] Brunet-Possenti F, Charpentier C, Collin G, Descamps D, Descamps V (2018). Impact of anti-interleukin-17 treatment on cutaneous and genital human papillomavirus infection. Br J Dermatol.

[21] Gosmann C, Mattarollo SR, Bridge JA, Frazer IH, Blumenthal A (2014). IL-17 suppresses immune effector functions in human papillomavirus-associated epithelial hyperplasia. J Immunol.

